# Canonical Non-Homologous End Joining in Mitosis Induces Genome Instability and Is Suppressed by M-phase-Specific Phosphorylation of XRCC4

**DOI:** 10.1371/journal.pgen.1004563

**Published:** 2014-08-28

**Authors:** Masahiro Terasawa, Akira Shinohara, Miki Shinohara

**Affiliations:** Institute for Protein Research, Osaka University, Osaka, Japan; Duke University, United States of America

## Abstract

DNA double-strand breaks (DSBs) can be repaired by one of two major pathways—non-homologous end-joining (NHEJ) and homologous recombination (HR)—depending on whether cells are in G1 or S/G2 phase, respectively. However, the mechanisms of DSB repair during M phase remain largely unclear. In this study, we demonstrate that transient treatment of M-phase cells with the chemotherapeutic topoisomerase inhibitor etoposide induced DSBs that were often associated with anaphase bridge formation and genome instability such as dicentric chromosomes. Although most of the DSBs were carried over into the next G1 phase, some were repaired during M phase. Both NHEJ and HR, in particular NHEJ, promoted anaphase-bridge formation, suggesting that these repair pathways can induce genome instability during M phase. On the other hand, C-terminal-binding protein interacting protein (CtIP) suppressed anaphase bridge formation, implying that CtIP function prevents genome instability during mitosis. We also observed M-phase-specific phosphorylation of XRCC4, a regulatory subunit of the ligase IV complex specialized for NHEJ. This phosphorylation required cyclin-dependent kinase (CDK) activity as well as polo-like kinase 1 (Plk1). A phosphorylation-defective XRCC4 mutant showed more efficient M-phase DSB repair accompanied with an increase in anaphase bridge formation. These results suggest that phosphorylation of XRCC4 suppresses DSB repair by modulating ligase IV function to prevent genome instability during M phase. Taken together, our results indicate that XRCC4 is required not only for the promotion of NHEJ during interphase but also for its M-phase-specific suppression of DSB repair.

## Author Summary

DNA double-strand breaks (DSBs) are highly toxic to cells and often lead to genome instability and cell death. Organisms have several DSB repair mechanisms to prevent such instability. Proper choice of DSB repair pathways is highly regulated during the cell cycle. Inappropriate choice of the DSB repair pathway often results in perturbation or failure of DSB repair, which is occasionally associated with tumorigenesis. Although the DSB repair pathways in the cell-cycle phases G1, S, and G2 are well elucidated, little is known about how cells deal with DSBs induced during M phase. We found that M-phase DSBs trigger massive chromosome aberrations, suggesting a lack of and/or inappropriate DSB repair during M phase. Notably, DNA damage response factors do not localize to mitotic chromosomes, and DSB repair pathways seem to be largely suppressed during M phase. In this study, we show that the efficiency of DSB repair is low during mitosis rather than being completely repressed. DSB repair, which generally prevents genome instability, causes genome instability during M phase. Cells have a mechanism to suppress DSB repair during M phase to prevent genome instability by modifying a non-homologous end-joining factor that is critical for DSB repair during other cell-cycle phases.

## Introduction

Double-strand breaks (DSBs) are one of the most consequential types of DNA damage. DSBs are usually repaired by one of two main repair pathways—canonical non-homologous end joining (C-NHEJ) or homologous recombination (HR) [1], [2]. Recently, however, a third less-characterized repair pathway, referred to as alternative NHEJ (A-NHEJ), was shown to play a critical role in DSB repair [3]–[7]. Once formed, DSBs are sensed by the Mre11-Rad50-Nbs1 (MRN) and Ku70–80 complexes, which recruit the ataxia-telangiectasia mutated protein (ATM) and DNA-dependent protein kinase catalytic subunit (DNA-PKcs) to the site [8]. ATM phosphorylates the C-terminus of histone H2AX to produce γH2AX [9]. The protein mediator of DNA damage checkpoint 1 (MDC1) recognizes γH2AX and is also phosphorylated by ATM [10]. Phosphorylated MDC1 then recruits the RING finger (RNF)-containing E3 ubiquitin ligase RNF8, which mediates ubiquitination of proteins at the damage site. Another E3 ubiquitin ligase, RNF168, recognizes RNF8 ubiquitination products and then ubiquitinates additional proteins. Eventually, this ubiquitination cascade leads to the recruitment of two main effector proteins, BRCA1 (breast cancer 1, early onset) and 53BP1 (p53-binding protein 1) to the DSB sites [11]–[15]. These effector proteins have opposite functions in DSB repair: BRCA1 leads to initiation of end resection to promote HR or A-NHEJ, whereas 53BP1 inhibits end resection to facilitate C-NHEJ [16], [17]. In the HR pathway, a DSB end sensed by the MRN complex is processed to introduce a 3′-overhanged single-stranded DNA end in a CtIP (C-terminal-binding protein interacting protein)-dependent manner. Subsequent recruitment of the single-stranded DNA binding protein, replication protein-A allows assembly of Rad51 recombinase filaments by Rad51 mediators such as BRCA2 and Rad51 paralogs including XRCC3 (X-ray repair cross-complementing group 3) to facilitate HR [2], [18]–[20].

Genome stability is assessed mainly during M phase, and failure of this process results in apoptosis or aneuploidy [21]. Despite the vast understanding of DSB repair in interphase, the molecular mechanisms underlying DSB repair during M phase are poorly understood. During M phase, DSBs induce γH2AX generation as well as recruitment of MDC1 and the MRN complex to DSB sites. The DNA damage response during M phase, however, prohibits the recruitment of RNF8, RNF168, BRCA1, or 53BP1 [22], [23]. RNF8 and 53BP1 recruitment and activities are inhibited through their M-phase specific phosphorylation [24] and PP4C/R3β phosphatase dephosphorylates 53BP1 in M to G1 transition [25]. Thus, DSB repair during mitosis appears to be mostly suppressed and to be regulated by mechanisms other than those active in other cell-cycle phases. Moreover, in contrast to the induction of interphase arrest/delay by DSBs, DNA damage induced by γ irradiation during M phase does not lead to substantial delay in mitotic exit, but instead it interferes with chromosome segregation and cytokinesis, and induces tetraploid G1 cells [26]. Some inhibitors of decatenation enzymes, such as topoisomerase II, induce metaphase arrest [27]. DNA damage alone, however, does not lead to metaphase arrest [28]. DSBs in nocodazole-arrested cells markedly reduce cell survival [22], indicating that cells cannot easily cope with DNA damage induced during M phase; little is known, however, about the mechanism and regulation.

We previously analyzed cell-cycle regulation of Lif1p (ortholog of human XRCC4), a regulatory subunit of the DNA ligase IV complex in *Saccharomyces cerevisiae* [29], [30]. DNA ligase IV is a NHEJ-specific DNA ligase that is essential for a final step of NHEJ. Phosphorylation of Lif1p during S/G2 and M phases results in a NHEJ mode switch from precise (C-NHEJ) to imprecise end joining [30]. NHEJ with imprecise end joining results in alterations in the DNA sequence after DSB repair [31]. Microhomology-mediated end joining (MMEJ) is one mechanism for imprecise end joining that is genetically distinguishable from C-NHEJ [32]. By contrast, A-NHEJ is roughly defined as NHEJ activity when core NHEJ factors (DNA ligase IV, Ku70 and Ku80) are inactivated. As MMEJ is also active in the absence of DNA ligase IV and Ku complexes, the terms A-NHEJ and MMEJ are sometimes used interchangeably in the literature [6]. In nocodazole-arrested cells, phosphorylation of Lif1p by a cyclin-dependent kinase (CDK) is important for imprecise end joining as DSB repair. The evolutionary conservation and biological significance of this regulatory of NHEJ mode switch is not well understood.

To clarify the influence of different mitosis-active DSB repair pathways on genome stability, we evaluated the formation of the anaphase bridges in M-phase cells defective for NHEJ, HR, or A-NHEJ. Anaphase bridges are a marker of genome instability [33], [34]. During M phase, cells appear to have mechanisms to control DSB repair and to prevent genome instability that are distinct from those in other cell-cycle stages.

## Results

### M-phase DSBs Introduced by Transient Etoposide Treatment Induce Anaphase Bridges

**Figure 1:**
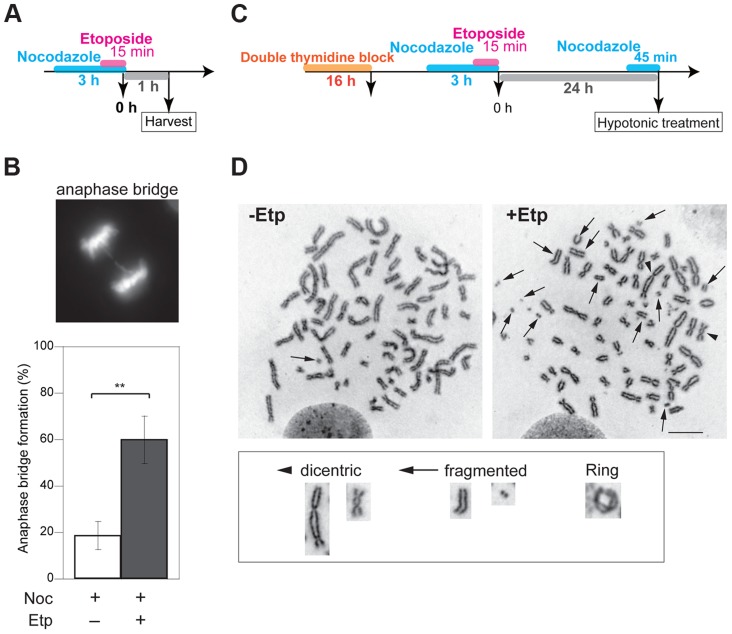
M-phase DSBs induce anaphase bridges and cause chromosome aberrations. (A) Procedure for induction of mitotic DSBs. (B) A typical image of an M-phase cell containing an anaphase bridge after M-phase DSB introduction (upper panel). Shown in bottom are anaphase bridge formation frequencies in etoposide-treated (gray bars) or non-treated cells (white bars) cells. Anaphase bridge formation frequency was calculated from the number of cells with anaphase bridges observed in total anaphase cells (≥50 for each experiment and condition). Error bars show the standard deviation from six independent experiments. Statistical significance was determined with the Student's t-test. ** *P*-value<0.01. (C) Procedure for mitotic DSB introduction for chromosome aberration analysis, which excludes non-M-phase cells. (D) Effect of etoposide on genomic instability of mitotic chromosomes. Shown are representative images of chromosome spreads from etoposide-treated cells (+Noc, +Etp) and non-treated cells (+Noc, −Etp). Arrows indicate fragmented chromosomes. Arrowheads show dicentric chromosomes. Small windows show representative images of typical dicentric chromosomes, fragmented chromosomes and ring chromosomes from etoposide-treated samples. Scale bar: 10 µm.

Formation of anaphase bridges often has been linked to chromosome instability because they promote abnormal chromosome segregation [33], [34]. Anaphase bridges have several causes, including DSBs, incomplete repair of DSBs, telomere dysfunction, and failure to decatenate intertwined sister chromatids after DNA replication [35]–[37]. Here we found that induction of DSBs by transient treatment with etoposide, a topoisomerase II inhibitor, during mitosis caused anaphase bridge formation. HeLaS3 cells were arrested at M phase by the addition of nocodazole, a microtubule polymerization inhibitor, and then treated transiently with etoposide (15 min) and fixed 1 h after nocodazole/etoposide release, when the majority of cells were in anaphase (Figure 1A). Bridge formation was examined by fluorescence microscopy. After release from nocodazole arrest, a substantial proportion of the non-etoposide-treated cells (19±6%; n = 3620) contained at least one anaphase bridge. The considerable high frequency of anaphase bridge formation without etoposide treatment was caused by nocodazole arrest (Figure S1A). In the etoposide-treated cells, however, the percentage of cells with anaphase bridges was significantly elevated (60±10%; n = 964; Figure 1B). Notably, the frequency of micronuclei formation increased in etoposide-treated cells (Table 1). Micronuclei often develop after formation of anaphase bridges [38]. In addition, the high frequency of anaphase bridge formation after M phase DSB induction was also observed in a different cell line, HCT116, a human colon carcinoma line (Figure S1B).

**Table 1:** Formation of micronuclei in etoposide-treated or non-treated cells.

	−Etp	+Etp	*P*-value^a^
Number of cells with micronuclei	21	35	0.015
Total number of cells	122	113	

a The significance of the difference between the non-treated (−Etp) and etoposide-treated (+Etp) cells was determined using Fisher's exact test.

We also tested another type of topoisomerase II inhibitor, ICRF-159, which inhibits topoisomerase II activity without induction of DSBs [39], [40]. Whereas continuous treatment of mitotic cells with ICRF-159 for 6 h led to anaphase bridges as reported [41], transient treatment did not affect the frequency of anaphase bridge formation (Figure S1C). Because topoisomerase II activity is important for decatenation and chromosome condensation in mitosis, we reasoned that its inhibition by etoposide is biologically relevant to an error in the action of topoisomerase II during normal mitosis. We also utilized other types of DNA damaging agents. Neocarzinostatin (NCS) is a radiomimetic DSB-inducing antitumor protein antibiotic [42], [43]. We observed that transient treatment of M-phase cells with NCS also increased formation anaphase bridges similar to what was seen in etoposide-treated cells (Figure S1C).

To examine the impact of etoposide treatment during mitosis on genome instability, we analyzed chromosome aberrations in metaphase spreads of 23 non-treated cells and 26 etoposide-treated cells at 24 h after transient treatment with etoposide (Figure 1C). A considerable proportion of the non-treated cells (35%) had fragmented chromosomes. In the etoposide-treated cells, however, we observed more fragmented chromosomes in all cells relative to control cells (Figure 1D, Table 2). We also identified other types of chromosome aberrations in the etoposide-treated cells, including dicentric and ring chromosomes. These aberrations are often seen in HR-defective cells, in which NHEJ may be inappropriately activated [44]. Dicentric chromosomes were observed in 62% of the etoposide-treated cells but never in the non-treated cells (Figure 1D, Table 2). Ring-shaped chromosomes, which appeared to be caused by inter-sister chromatid fusion, were observed in 15% of the etoposide-treated cells. We also observed similar types of chromosome aberrations are increased in etoposide-treated HCT116 cells (Table S1). These results indicated that etoposide-induced DSBs in M-phase lead to genome instability.

**Table 2:** Chromosome aberration analysis.

	−Etp	+Etp	*P*-value^a^
Fragmented chromosomes	8	26	<0.001
Ring chromosomes	0	4	0.11
Dicentric chromosomes	0	16	<0.001
Metaphase cells analyzed (N)	23	26	

a The significance of each difference between the non-treated (−Etp) and etoposide-treated (+Etp) cells was determined using Fisher's exact test.

### DSBs Introduced in Mitosis Are Partially Repaired during Mitosis

**Figure 2:**
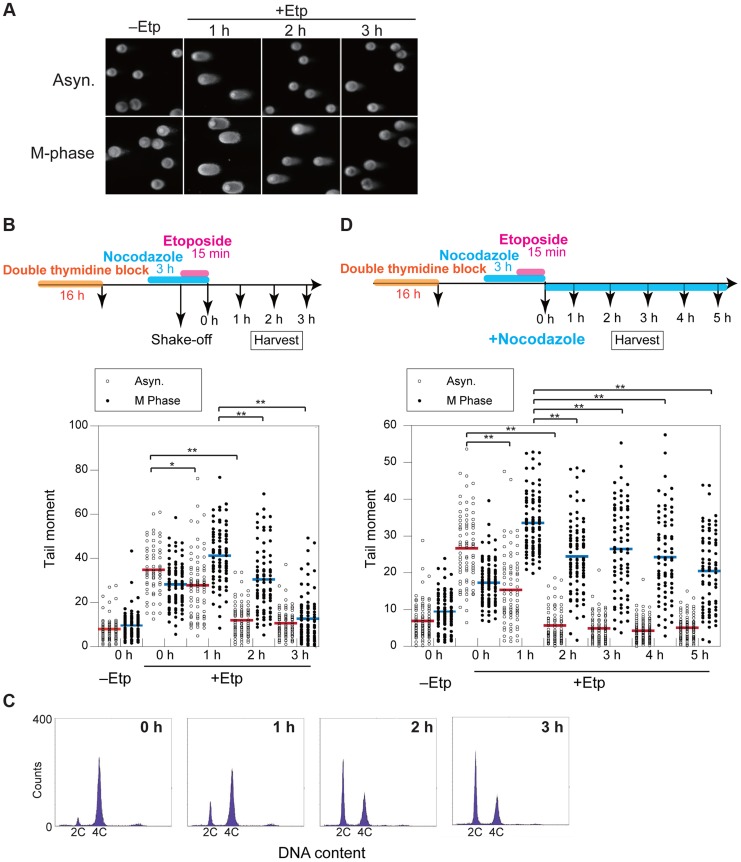
DSB repair occurs with low efficiency during mitosis. (A) Asynchronous cells (Asyn.) or nocodazole-arrested (M-phase) cells were analyzed by the neutral comet assay. Typical images are shown for non-treated (−Etp) or etoposide-treated (+Etp) cells at each time point after release from etoposide treatment. (B) Comparison of DSB repair kinetics between mitotic and asynchronous cells. Shown is the comet assay procedure for detecting transiently introduced M-phase DSBs (upper figure). Shown is the distribution of tail-moment values in the non-treated cells (−Etp) or DSB-induced cells (+Etp) in the asynchronous condition (open circles) or nocodazole arrested condition (closed circles) at each time point after release from etoposide treatment. Red and blue bars show average values of tail moments for each condition. Statistical significance was analyzed using Dunnett's test. ** *P*-value<0.01; * *P*-value<0.05. (C) Cell-cycle distribution of cells with DSBs after nocodazole arrest. Cells arrested with nocodazole were treated to induce DSBs and were used for the comet assay. Cells were analyzed for DNA content by FACS at the indicated times after release from etoposide treatment. The *x*-axis values correspond to DNA content. (D) Procedure for mitotic DSB introduction by the comet assay during constitutive M-phase arrest (upper scheme). Shown is DSB repair kinetics in prometaphase-arrested cells by comet assay. Distribution of tail moment values under the continuous nocodazole-arrested condition is shown graphically. Statistical significance was analyzed using Dunnett's test. ** *P*-value<0.01.

To clarify the kinetics of DSB formation and repair during mitosis, we used the neutral comet assay to quantify the total amount of DSBs in M-phase-arrested and asynchronous cells at several time points after DSB introduction. Mitotic cells at 1 h after etoposide treatment showed comet tailing, indicating that the treatment induced DSBs on chromosomes (Figure 2A). Because the extent of tailing correlates with the occurrence of DSBs, we could establish the kinetics of DSB repair in the asynchronous and mitotic cells by quantifying the extent and intensity of the tail over time (Figure 2B). To more thoroughly enrich for mitotic cells, cells were arrested at early S phase by double thymidine block, released into the cell cycle, and then rearrested with nocodazole. In addition, mitotic cells were collected by the shake-off method prior to etoposide treatment (Figure 2B). In asynchronous cells, the maximum occurrence of DSB was observed immediately after release from etoposide treatment, and the DSB signal decreased to basal level within 2 h. In mitotic cells, however, the occurrence of DSBs reached a maximum at 1 h after release from etoposide treatment and then returned to basal level at 3 h (Figure 2B). This result suggested that DSB repair in mitosis was slower than during other cell-cycle stages. Fluorescence-activated cell sorting (FACS) of M-phase-arrested cells at 2 h after etoposide release revealed that 48% of the cells had already exited mitosis and entered G1 (Figure 2C), suggesting that most DSBs formed in M phase could be carried over and repaired in the next G1 phase. Moreover, we investigated 53BP1 localization in the cells as a marker of DNA lesions in G1 because 53BP1 promotes NHEJ in G1 [22]. We compared 53BP1 staining at 0, 1, and 2 h after etoposide treatment and found that the 53BP1 foci that co-localized with γH2AX signals, as a DSB marker [9] (Figures S2C and D). These 53BP1 foci appeared only in interphase nuclei and were observed in 32% of cells at 2 h, when half of cells were in G1 as revealed by FACS (Figure 2C). This finding suggested that DNA damage induced during mitosis was not completely repaired during mitosis; rather, the DSBs were presumably repaired in the subsequent G1.

We next observed the localization of representative HR-specific and NHEJ-specific factors on mitotic chromosomes after induction of DSBs by etoposide to determine whether DSB repair pathways are active during mitosis in HeLaS3 cells. Although γH2AX foci were observed on mitotic chromosomes 1 h after etoposide treatment, Rad51 and 53BP1 did not localize to mitotic chromosomes (Figures S2A–D). These results confirmed previous results [22], [23], supporting the idea that both HR and NHEJ are essentially suppressed during mitosis.

To determine whether DSB repair either does not occur during mitosis or is just inefficient, we assessed at DSB repair under continuous nocodazole arrest after etoposide treatment of mitotic cells (Figure 2D). DSBs reached a maximum at 1 h after etoposide treatment, and the DSB signals gradually decreased upon further incubation with nocodazole. After 5 h, the occurrence of DSBs decreased to 60.9% of the maximum (Figure 2D). Although the efficiency of DSB repair during mitosis was much lower than in asynchronous cells, a substantial proportion of the DSBs were repaired during mitosis.

### Anaphase Bridges Are Promoted by NHEJ Factors but Suppressed by CtIP

**Figure 3:**
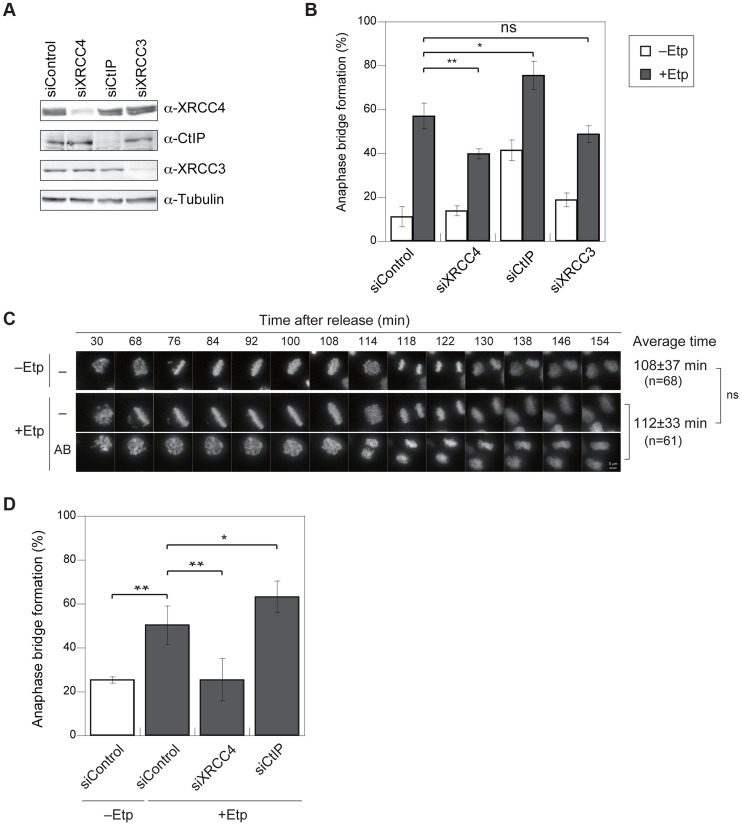
Anaphase bridges are formed by NHEJ and HR and are inhibited by CtIP-dependent end resection. (A) Representative western blots showing the siRNA-mediated depletion of XRCC4, CtIP, or XRCC3 in HeLaS3 cells as well as control siRNA-treated cells. (B) Frequency of anaphase bridge formation in HeLaS3 cells transfected with an siRNA targeting XRCC4, CtIP, or XRCC3 (or control siRNA) with (+Etp) or without (−Etp) induction of mitotic DSBs by etoposide. Anaphase cells (≥50) were scored for each experiment and condition. Error bars show the standard deviation from three independent experiments. Statistical significance was analyzed with the Student's t-test. ** *P*-value<0.01, ns, not significant. (C) Anaphase bridge formation was observed with time-lapse live-cell imaging of a typical HeLa cell at the indicated time after release from etoposide treatment with (AB) or without (−) anaphase bridge formation. In addition, shown is an average of duration when required to transit to anaphase in non-treated (−Etp) or etoposide-treated (+Etp) cells with numbers of cells analyzed. Statistical significance was analyzed with the Student's t-test. Scale bar, 5 µm; ns, not significant. (D) Anaphase-bridge formation observed by time-lapse imaging after DSB induction in the indicated knockdown cells. Prometaphase cells (≥25) were chosen and analyzed for anaphase bridge formation for each experiment. Error bars show standard deviation from 3 to 11 independent experiments. Statistical significance was analyzed with the Student's *t*-test. ** *P*-value<0.01, * *P*-value<0.05.

We hypothesized that anaphase bridges could be formed by inappropriate activation of DSB repair during mitosis. To determine which DSB repair pathway is involved in bridge formation, we examined the effect of small interfering RNA (siRNA)-mediated knockdown of XRCC4 (an NHEJ factor), CtIP (a HR- and A-NHEJ-associated end resection factor), or XRCC3 (an HR-specific factor) on anaphase bridge formation. Western blotting confirmed that siRNA treatment efficiently decreased the endogenous proteins at 72 h after transfection (Figure 3A). The knockdown cell lines were arrested at M phase and then transiently treated with etoposide. The frequency of anaphase bridge formation at 1 h post-treatment was 57.1±6% (n = 488) in negative control siRNA–transfected cells (Figure 3B). By comparison, the percentage of cells containing anaphase bridges was significantly lower in XRCC4-knockdown cells (40±2%, n = 565), and XRCC3-knockdown cells showed a slight decrease in bridge formation (49±4%, n = 403), but the decrease in the latter case was not significantly different from control cells (Figure 3B). Anaphase bridge formation was significantly higher in CtIP-knockdown cells (76±6%, n = 385). On the other hand, CtIP-knockdown cells showed a high frequency of anaphase bridge formation after nocodazole arrest even without etoposide-treatment (Figure 3B), so the difference between negative control and CtIP-knockdown cells was not significant when we compared each value after subtraction of anaphase bridge frequency without etoposide treatment (Figure S3A). This result cast doubts on the possibility that the high frequency of anaphase bridge formation in CtIP-knockdown cells might be caused indirectly by a non-M-phase event.

To eliminate the possibility that a non-M-phase cell fraction contributed to anaphase bridge formation in the above bulk assay, we monitored bridge formation in live cells using GFP-histone H2B–expressing cells with or without M-phase DSBs (Figure 3C). We concentrated M-phase cells using plural methods (see Materials and Methods) and chose prometaphase cells under a microscope for the live imaging analysis and took time-lapse images every 2 min for 3 h. First, we compared the time spent transiting from prometaphase (arrest-point) to anaphase in non-treated and etoposide-treated cells. This transition took 108±37 min in non-treated cells and 112±33 min in etoposide-treated cells, and the difference was not significant (Figure 3C), indicating that transient etoposide treatment did not affect the prometaphase-to-anaphase transition. Next, we analyzed anaphase bridge formation (Figure 3D). Similar to our observations in bulk assay, the frequency of anaphase bridge formation in DSB-induced M-phase cells was significantly higher (50±9%, n = 458) than in the control (26±1%, n = 157). Analysis of each knockdown cell line confirmed that bridge formation in DSB-induced M-phase cells was significantly reduced in XRCC4-knockdown cells (26±10%, n = 268). By contrast, anaphase bridge formation increased significantly in the CtIP-knockdown cells (63±7%, n = 191; Figure 3D). In contrast to the results from our bulk assay, there was not a significant difference between the negative control and the CtIP-knockdown cells that had not been treated with etoposide (Figure S3C). These results indicated that both the NHEJ and HR pathways, especially NHEJ, promote anaphase bridge formation and that CtIP prevents anaphase bridge formation when DSBs are introduced in mitotic chromosomes.

### XRCC4 Is Phosphorylated during Mitosis

**Figure 4:**
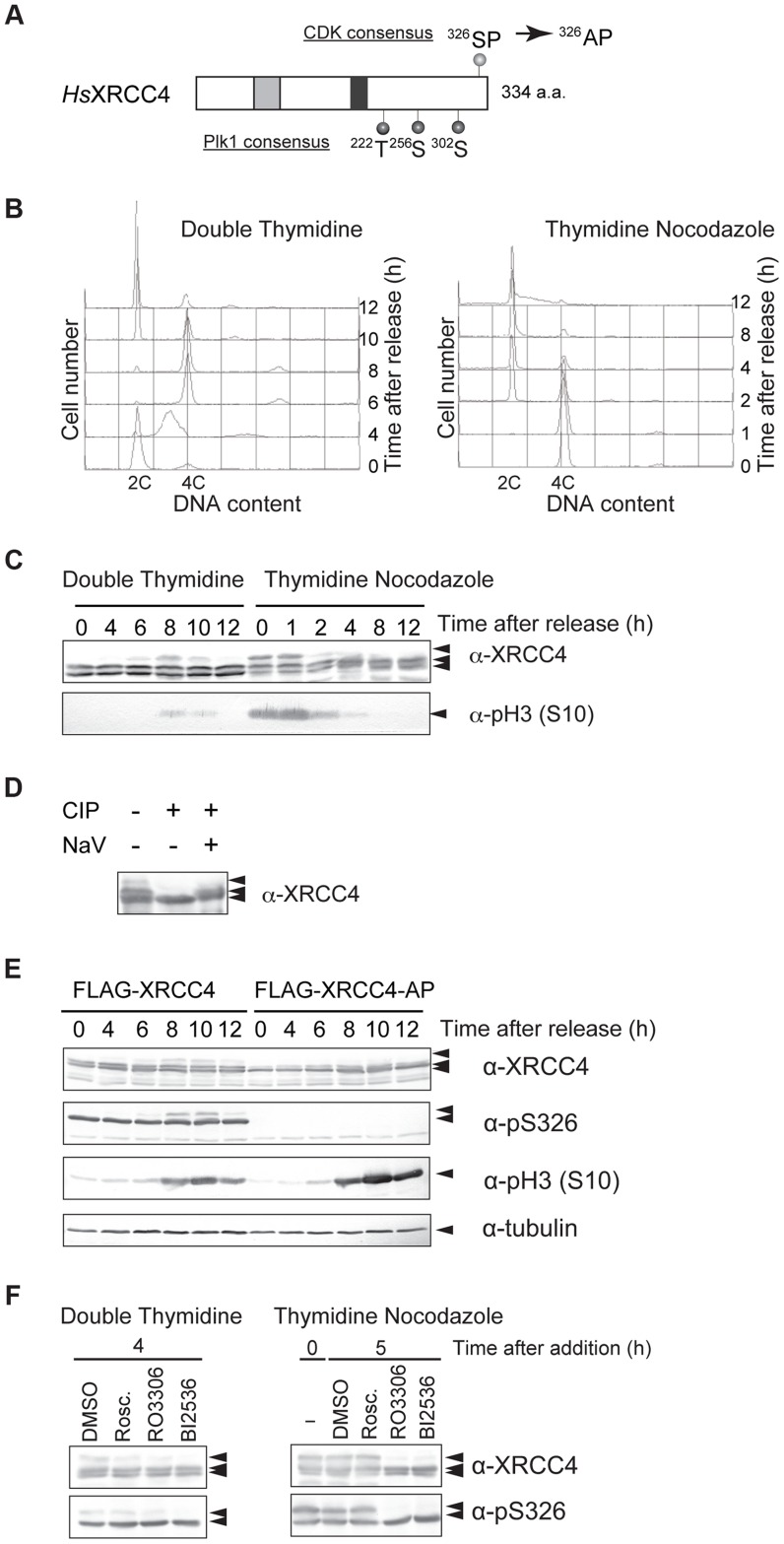
M-phase specific phosphorylation of XRCC4. (A) Domain structure of XRCC4. Serine 326 near the C-terminus of human XRCC4 is a potential CDK phosphorylation site. Threonine 222, serine 256 and serine 302 are potential Plk1 phosphorylation sites. Substitution of serine 326 with alanine results in the mutant XRCC4-AP. The gray box and black box indicate XLF- and DNA ligase IV–binding sites, respectively. (B) Cell-cycle progression after release from double thymidine or thymidine-nocodazole block. Cells were arrested in G1/S via double thymidine or thymidine-nocodazole blocks and were then released to resume the cell cycle. Cells were harvested at the indicated times, lysed with Triton X-100, and analyzed for DNA content by FACS. (C) Western blot of lysates from HeLaS3 cells after release from double thymidine block or thymidine-nocodazole block. Cells were harvested at the indicated times, lysed in SDS-PAGE loading buffer, and subjected to western blotting. Each membrane was incubated with anti-XRCC4 or anti-phosphorylated histone H3 (S10), a marker for M phase. (D) Effect of phosphatase treatment on XRCC4 modification. FLAG-tagged XRCC4 was immunoprecipitated from asynchronous HeLaS3 cells expressing FLAG-tagged XRCC4. The immunoprecipitate was treated with calf intestine phosphatase (CIP) in the absence or presence of its inhibitor, NaV. (E) Western blot of lysates from FLAG-tagged XRCC4 and FLAG-tagged XRCC4-AP cells after release from double thymidine block. FLAG-tagged XRCC4 and FLAG-tagged XRCC4-AP cells were harvested at the indicated times, lysed in SDS-PAGE loading buffer, and analyzed by western blotting with anti-XRCC4, anti-phosphorylated serine 326 of XRCC4 (anti-pS326), anti-phosphorylated histone H3 (S10) (a marker for M phase), or anti-α-tubulin (internal control). (F) Effect of a CDK and Plk1 inhibitors on phosphorylation of XRCC4. Cells were arrested by double thymidine block. Cells were released into the cell cycle (double thymidine) or treated with nocodazole continuously (thymidine-nocodazole), and then treated with the CDK inhibitor roscovitine (20 µM; Rosc.) or RO3306 (20 µM) or the Plk1 inhibitor BI2536 (200 nM) for 4 h or 5 h, respectively. XRCC4 was analyzed by western blotting with anti-XRCC4 or anti-pS326.

XRCC4 has a CDK phosphorylation consensus site near its C-terminus. From our previous study of CDK-dependent phosphorylation of Lif1 [30], we hypothesized that XRCC4 is also post-translationally modified/regulated during the cell cycle. To determine whether XRCC4 is modified in a cell cycle–dependent manner, HeLaS3 cells were arrested at the G1/S boundary by double thymidine block and then released into the cell cycle. Cell-cycle progression was monitored by FACS (Figure 4B). Cells were harvested at different times, and cell lysates were subject to western blotting with an antibody against XRCC4. Two XRCC4 bands were detected throughout the cell cycle, and an additional slower migrating band was detected at 6, 8 and 10 h, which roughly corresponded to M phase (Figure 4C, left). Thymidine-nocodazole-arrested cells were similarly analyzed, and we found that mitotic cells contained the additional slow-migrating XRCC4 band in the 0 and 1 h samples in addition to the two constant bands (Figure 4C, right). These results suggested that this modification of XRCC4 occurs specifically during mitosis and disappears in the next G1.

To confirm the cell-cycle timing of XRCC4 modification, we performed western blotting with an antibody against phosphorylated histone H3S10, a mitotic marker. We observed phosphorylated H3S10 in samples from 8 or 10 h after release from double thymidine block and in samples from 0, 1 or 2 h after release from thymidine-nocodazole block (Figure 4C). The timing of H3S10 coincided with the appearance of the slower migrating XRCC4 band, confirming that XRCC4 is indeed modified during M phase.

To determine whether the modification of XRCC4 indeed reflected phosphorylation, FLAG-tagged XRCC4 from asynchronous cells was immunoprecipitated and treated with calf intestinal phosphatase in the presence or absence of phosphatase inhibitor (Figure 4D). Treatment with the phosphatase abolished the two slow-migrating XRCC4 bands, indicating that these bands were likely phosphorylated XRCC4. Taken together, the results indicated that XRCC4 is phosphorylated specifically during mitosis.

### CDK1 and Plk1 Mediates M-phase–Specific Phosphorylation of XRCC4

To assess whether CDKs are involved in the cell cycle–specific phosphorylation of XRCC4, we established a cell line that expressed FLAG-tagged XRCC4 with a mutation in the putative CDK phosphorylation motif (XRCC4-AP, serine 326 substituted with alanine; Figure 4A). We then examined the effect of this mutation on the modification of XRCC4. FLAG-tagged XRCC4 and XRCC4-AP cells were arrested at the G1/S boundary with double thymidine block and then released into the cell cycle. Cells were harvested at various times and analyzed by western blotting with anti-XRCC4. As FLAG-conjugated XRCC4 migrates slower than endogenous XRCC4, we could distinguish FLAG-tagged XRCC4 or XRCC4-AP on the blot (Figure S4A). As with the untagged protein, in FLAG-XRCC4-expressing cells, two bands were detected throughout the cell cycle, and an additional M phase-specific slower migrating band appeared at 8 to 12 h after the release (Figure 4E). In FLAG-XRCC4-AP-expressing cells, however, the both slower migrating bands almost disappeared, indicating that the S326 contributed to the formation of the multiple slower migrating XRCC4 bands. In addition, we examined phosphorylation at S326 using an antibody that recognizes phospho-S326 of XRCC4 (anti-pS326, Figure 4E). In FLAG-XRCC4-expressing cells, both slower migrating bands, including the M-phase-specific band, but not the faster migrating band were recognized by anti-pS326; in FLAG-XRCC4-AP-expressing cells, however, no anti-pS326 specific XRCC4 signal was detected. These results strongly suggest that S326 is phosphorylated *in vivo*.

To determine whether phosphorylation of XRCC4 at S326 depends on CDK activity, we treated HeLaS3 cells with the CDK inhibitor roscovitine or CDK1 inhibitor RO3306. Cells were arrested at the G1/S boundary with double thymidine and then released. Cells were incubated with or without roscovitine or RO3306, harvested at 8 h after release from double thymidine block, and endogenous XRCC4 was analyzed by western blotting. We found that the slower migrating band was diminished at 8 h when cells were treated with roscovitine or particularly with RO3306, but not with DMSO alone (Figure 4F). The M-phase specific slower migrating band was also diminished by treatment with RO3306 in mitotic cells arrested by thymidine nocodazole block (Figure 4F). In contrast to the yeast Lif1, however, the CDK phosphorylation–site of XRCC4 overlaps with the polo box core domain consensus ([S]-[pS/pT]-[P/X]) [45]. Actually, XRCC4 has three potential polo-like kinase 1 (Plk1) phosphorylation target sites ([D/E]-[X]-[S/T]-[Φ]) (Figure 4A) [46]. We also looked at involvement of the M-phase kinase, Plk1, in this phosphorylation. Treatment of cells with BI2536, a Plk1 inhibitor, abolished the mitosis-specific slower phosphorylation signal in both cells released from double thymidine block and cells arrested by thymidine nocodazole (Figure 4F). These results suggested that both CDK1 and Plk1 activities are responsible for the observed phosphorylation of XRCC4 at multiple sites during M-phase. In addition, we examined phosphorylation at S326 by using pS326 antibody in the presence of those inhibitors. We found that the mitosis-specific slower migrated XRCC4 signal disappeared by treatment with RO3306 or BI2636. In contrast, the other phosphorylated signal, which was abundant throughout the cell cycle, were not affected by CDK as well as Plk1 inhibitor-treatment. This indicates that CDKs and Plk1 do not play a major role in the phosphorylation at S326.

### DNA Ligase IV, but Not XRCC4, Localizes to Mitotic Chromosomes

The DNA ligase IV complex is required for the final step of C-NHEJ; the ligase forms a complex with XRCC4 and XRCC4-like factor (XLF) that is important for the activity of the DNA ligase IV [47], [48]. We examined the subcellular localization of the DNA ligase IV complex components with immunostaining and found no difference in localization between non-treated and etoposide-treated cells (Figure S4B). In interphase cells, both DNA ligase IV and XRCC4 localized to the nucleus. During mitosis, however, DNA ligase IV localized to mitotic chromosomes, whereas little XRCC4 localized to chromatin but rather mainly localized to the cytoplasm (Figure S4B), as reported [49]. Because direct interactions between DNA ligase IV and XRCC4 are essential for DNA ligase IV complex activity, it is likely that the activity of the complex was dramatically reduced during mitosis.

**Figure 5:**
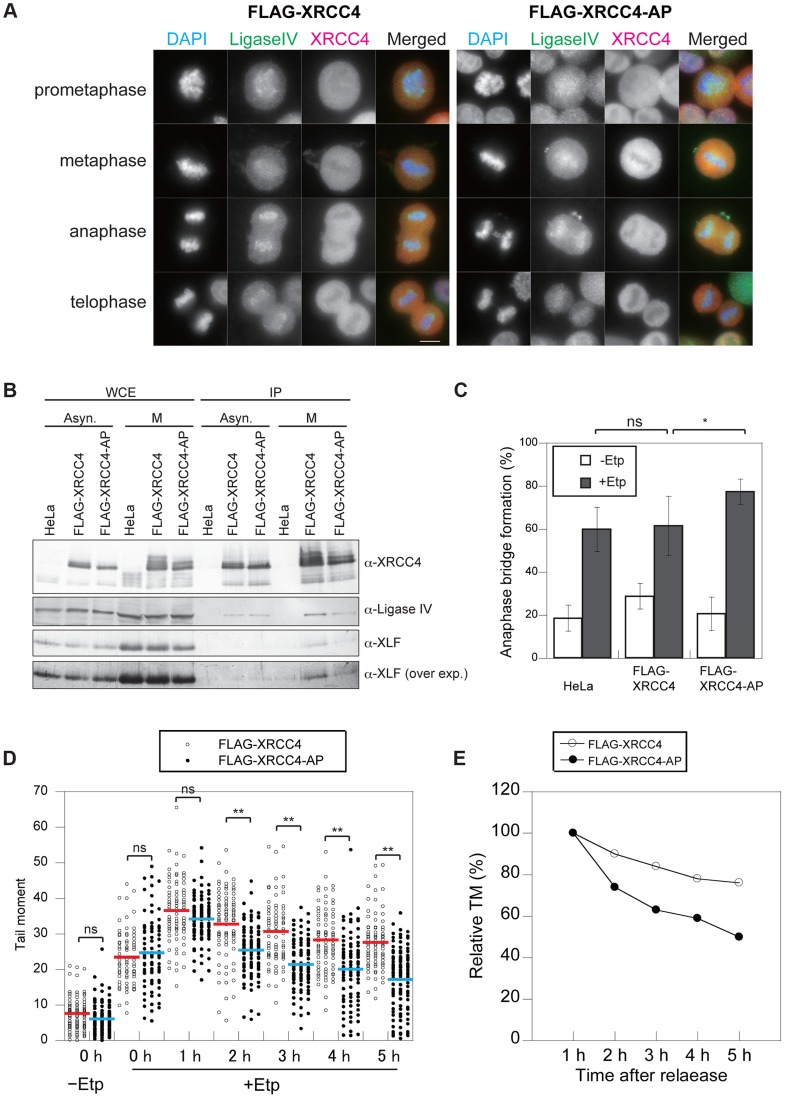
Phosphorylation of XRCC4 at S326 is involved in suppression of DSB repair during M-phase. (A) Staining of DNA ligase IV (green; anti-DNA ligase IV) and XRCC4 (red; anti-XRCC4) in etoposide-treated cells. Indirect immunostaining was performed as described in Materials and Methods for FLAG-XRCC4- or FLAG-XRCC4-AP-expressing cells at 1 h after release from nocodazole arrest. Shown is an image for each mitotic phase. Scale bar, 10 µm. (B) Co-immunoprecipitation of DNA ligase IV and XLF with FLAG-tagged XRCC4 or FLAG-tagged XRCC4-AP. Asynchronous cells (Asyn.) and M-phase arrested cells (M) were immunoprecipitated with anti-FLAG, and then subjected to western blotting using antibody against XRCC4, DNA ligase IV, and XLF. An overexposure image (over exp.) of the same western blot membrane using anti-XLF is shown at the bottom. (C) Frequency of anaphase bridge formation in HeLaS3 cells with control siRNA or in XRCC4-depleted, FLAG-XRCC4-expressing, and FLAG-XRCC4-AP-expressing cells. Shown are anaphase bridge formation frequencies in etoposide-treated (gray bars) or non-treated cells (white bars) of each cell line. Anaphase cells (≥50) were analyzed for each experiment and condition. Error bars show the standard deviation from seven independent experiments. Statistical significance was analyzed with the Student's t-test. * *P*-value<0.05; ns, not significant. (D) Detection of DSB repair kinetics in XRCC4 and XRCC4-AP cells. Shown is the distribution of tail moment (TM) values under the continuous nocodazole-arrested condition in DSB-induced FLAG-XRCC4-expressing cells (XRCC4; open circles) or FLAG-XRCC4-AP-expressing cells (closed circles) with (+Etp) or without (−Etp) etoposide at the indicated time points after release from etoposide treatment. Statistical significance was analyzed with the Student's t-test. ** *P*-value<0.01; ns, not significant. (E) Detection of DSB repair kinetics in XRCC4-depleted, FLAG-XRCC4-expressing (open circles), and FLAG-XRCC4-AP-expressing (closed circles) cells. Shown are kinetics of average relative TM values in etoposide-treated cells of each cell line. Relative TM values were calculated by dividing each TM value by the average peak TM value at 1 h.

We next examined whether the phosphorylation of XRCC4 is involved in its failure to localize to mitotic chromosomes. We analyzed the localization of FLAG-XRCC4-AP and DNA ligase IV on mitotic chromosomes after M-phase DSB induction. As with the wild-type protein, FLAG-XRCC4-AP did not localize to mitotic chromosomes, whereas DNA ligase IV localized to mitotic chromosomes (Figure 5A). By immunoprecipitation, we observed that both wild-type FLAG-XRCC4 and FLAG-XRCC4-AP mutant proteins interacted with DNA ligase IV and XLF in both asynchronous and M-phase cells (Figure 5B). These results suggested that M-phase specific phosphorylation of XRCC4 is not involved in regulating XRCC4 subcellular localization and DNA ligase IV complex formation.

### Phosphorylation at S326 of XRCC4 Prevents Anaphase Bridge Formation

To investigate the role of phosphorylation at S326 of XRCC4, we analyzed anaphase bridge formation in XRCC4-AP cells (Figure 5C). We designed siRNA-resistant FLAG-tagged XRCC4 and FLAG-tagged XRCC4-AP constructs to be expressed in a strain in which the endogenous XRCC4 was depleted with siRNA (Figure S4A). After the depletion of endogenous XRCC4, the cells were arrested at M phase, transiently treated by etoposide, released into the cell cycle for 1 h, and then harvested and fixed. The percentage of cells containing anaphase bridges increased to 62±14% (n = 1068) in the FLAG-XRCC4 cells after etoposide treatment, which was not significantly higher than in non-XRCC4-depleted cells (HeLaS3 cell with control siRNA). By contrast, the frequency of cells containing anaphase bridges was significantly higher in the XRCC4-AP cells (78±6%, n = 1473; Figure 5C and S3B). These results suggested that phosphorylation of XRCC4 during mitosis prevents the formation of anaphase bridges.

We next examined whether the phosphorylation-defective mutant XRCC4-AP affected the efficiency of DSB repair during M phase. Both FLAG-XRCC4 and FLAG-XRCC4-AP cells (each of which was depleted of endogenous XRCC4) were arrested at M phase, and then we assessed the efficiency of DSB repair under continuous nocodazole arrest after etoposide treatment (Figure 2D). Similar to M-phase-arrested HeLaS3 cells, DSBs were gradually reduced in FLAG-XRCC4 cells. In XRCC4-AP cells, however, statistically significant decreases in comet tail moments compared with FLAG-XRCC4 cells were observed at 2 h and later time points, demonstrating that DSB repair in XRCC4-AP cells was more rapid than in FLAG-XRCC4 cells (Figures 5D and E, Figures S5A and B). This result indicated that phosphorylation of XRCC4 during mitosis inhibited DSB repair and that this function is important for preventing chromosome instability.

## Discussion

We established a method to introduce DSBs specifically and efficiently in mitotic chromosomes by transient treatment with etoposide in nocodazole-arrested cells. Using this method, we found that mitotic DSBs induce severe chromosome aberrations such as dicentric and fragmented chromosomes, which may be initiated by anaphase bridges. These results suggest that there is a process to connect sister or individual chromosomes in response to DSBs formed during M phase. DNA ligase IV–dependent C-NHEJ contributes to dicentric chromosome formation by telomere fusion in cells with dysfunctional telomeres [50], and it is thought that the dysfunctional telomeres act as DSB ends [51]. Taken together with our observation of elevated numbers of dicentric chromosomes, these findings suggest that NHEJ may be involved in the development of chromosome aberrations from mitotic DSBs. This metabolism of mitotic DSBs might explain the molecular mechanism of etoposide-induced secondary leukemia with chromosomal translocations in the gene for mixed-lineage leukemia/myeloid lymphoid leukemia that is stimulated by failure of the G2/M checkpoint [52].

Topoisomerase II is essential for chromosome condensation as well as chromosome decatenation during mitosis [53], [54]. Although it is reasonable to conclude that chromosome aberrations we observed after etoposide treatment were caused by the defects in chromosome condensation or decatenation, transient treatment with the topoisomerase II catalytic inhibitor ICRF159 did not promote anaphase bridge formation (Figure S1C). This result indicates that chromosomal events induced by etoposide are not due to general loss of topoisomerase II activity. This result suggests that DSBs are responsible for the observed increase of anaphase bridge formation in our system.

The fact that XRCC4 knockdown reduced anaphase bridge formation also suggests that NHEJ contributes to the formation of some of these bridges during mitosis. On the other hand, CtIP knockdown increased anaphase bridge formation after mitotic DSB induction, suggesting that another repair pathway(s) represses chromosome rearrangement during M phase. Etoposide treatment accumulates covalent bound of topoisomerase II at the DSB ends. It is reported that catalytic activity of CtIP is required for the removal of adducts such as topoisomerase II from the DSB sites, and is distinguishable from HR activity *in vivo* [55]. In our case, not only the etoposide, but also the DSB-inducing reagent neocarzinostatin increased anaphase bridge formation in CtIP-knockdown cells (Figure S1D), implying that the CtIP dependency was not caused by covalent attachment of topoisomerase II at DSB ends in etoposide-treated cells. Since cells not treated with etoposide showed an increase in anaphase bridge formation in CtIP-knockdown cells (Figure 3B), we could not exclude the possibility that the anaphase bridges originated from non-M-phase events, such as replication stress, resulting in an increase over the basal level in CtIP-knockdown cells (Figure S3A). However, we did not observe a significant increase in anaphase bridge formation frequency without etoposide treatment in CtIP-knockdown M-phase cells that were chosen based on their morphology as viewed after mitotic shake-off (Figure S3C, Materials and Methods). CtIP is involved in DSB-end resection in the HR and A-NHEJ pathways [56], and therefore our results imply that DSB-end resection is required for the repair of mitotic DSBs. The HR-specific factor XRCC3, however, was not found to be critical for the suppression of anaphase bridges (Figure 3B), indicating that, of the two major DSB-repair pathways, NHEJ is the more toxic to mitotic cells than HR. We also observed that 53BP1 and Rad51 were not recruited to M-phase-induced DSB sites on mitotic chromosomes or to anaphase bridges. Moreover, artificial activation of 53BP1 during M phase promotes the formation of dicentric chromosomes through telomere fusions [24], and it is well established that NHEJ activity is critical for telomere-telomere fusion [57]. Taken together, these studies strongly suggest that inappropriate activation of the NHEJ pathway causes chromosome bridges, which lead to chromosome aberrations in the next cell cycle.

In this study, we detected a significant reduction of DSBs in cells continuously arrested in prometaphase (Figure 2D), and we found that CtIP is involved in the repression of anaphase bridge formation during mitosis (Figure 3B). These results suggest that the A-NHEJ pathway, initiated by CtIP-dependent DSB-end resection, might be involved in anaphase bridge suppression and/or the repair the M-phase DSBs, which somehow may suppress chromosome aberrations during M phase. On the other hand, a large proportion of cells with M-phase-induced DSBs entered G1 and reacquired 53BP1 foci at 2 h after etoposide treatment (Figures 2B and C, Figures S2C and D). Moreover, the timing of transition from prometaphase to anaphase of cells with DSBs was indistinguishable from that of cells without DSBs (Figure 3C), suggesting that transient etoposide-induced M-phase DSBs do not induce M-phase delay by the activation of the DNA damage response pathway, consistent with a previous report [22]. Thus our observations are distinguishable from the M-phase arrest caused by topoisomerase-inhibitor-induced decatenation defects [28]. Based on our results, we propose that mitotic DSB repair is completed in G1 primarily by C-NHEJ, but that A-NHEJ, which is mediated by annealing via microhomology in single-stranded DNA regions, might contribute to bridging DSB ends to prevent fragmentation during chromosome segregation.

We found that both mitosis-specific phosphorylation of XRCC4 as well as its phosphorylation throughout the cell cycle were substantially reduced by introduction of an amino acid substitution at S326 (Figure 4E). Actually, a phosphorylation at the S326 resides within the polo box recognition motif [45]. A previous phosphoproteomics study revealed that S256 as well as S326 is phosphorylated during mitosis [58]. Because S256 resides within the Plk1 phosphorylation target motif (Figure 4A), it is possible that phosphorylation at S326 may prime XRCC4 for subsequent Plk1-mediated phosphorylation during mitosis, which produces multiple electrophoretically retarded XRCC4 bands (Figure 4C). This hypothesis is supported by our result that treatment with not only a CDK1 inhibitor but also a Plk1 inhibitor affected the appearance of the M-phase-specific slower migrating band (Figure 4F). On the other hand, the phosphorylation throughout the cell cycle was not affected by treatment with CDK inhibitors (Figure 4F). This suggests that other unknown kinase(s) contributes phosphorylation at S326 of XRCC4.

We demonstrated that the S326-dependent phosphorylation(s) of XRCC4 contributes to the suppression of anaphase bridge formation through repression of DSB repair during M-phase (Figures 5C–E). XRCC4 did not localize to mitotic chromosomes, whereas DNA ligase IV was specifically recruited to mitotic chromosomes [49]. We showed that XRCC4 phosphorylation is not responsible for the failure of XRCC4 to localize to mitotic chromosomes and also does not affect the localization of DNA ligase IV to mitotic chromosomes (Figure 5A). In addition, XRCC4 phosphorylation did not affect DNA ligase IV complex formation during mitosis (Figure 5B). So far, molecular function of the XRCC4 phosphorylation in repression of DSB repair during M-phase is still unknown. It was reported that 53BP1 activity, which is required for promotion of NHEJ prior to XRCC4, is also repressed by mitosis-specific phosphorylation of 53BP1 as well as RNF8 via CDK1 and Plk1. Interestingly, as in the case of XRCC4, combination of phosphorylation-defective mutations of 53BP1 and RNF8 showed restoration of DNA repair during M-phase [24]. Combining this report and our findings, there should be multiple inhibition mechanisms to shut off toxic NHEJ during M-phase through M-phase specific phosphorylation of NHEJ factors mediated by CDK1 and Plk1.

Lif1, which is the *S. cerevisiae* ortholog of XRCC4, is phosphorylated by CDKs from S to M phase, and this phosphorylation is involved in NHEJ in G2/M-arrested cells, but not in G1 cells. Lif1 phosphorylation plays a role in suppressing C-NHEJ during S to M-phase through a pathway that is dependent on Sae2, the *S. cerevisiae* ortholog of CtIP [30]. If the function of CDK-dependent phosphorylation of Lif1 is conserved in humans, then mitotic XRCC4 phosphorylation might be involved in suppressing C-NHEJ to prevent chromosome instability in human cells via CtIP function when mitotic DSBs are introduced. This possibility is supported by our observation that rapid repair of M-phase DSBs is associated with more anaphase bridges in XRCC4-AP cells. In summary, XRCC4, as a regulatory subunit of the DNA ligase IV complex, is required not only for C-NHEJ in interphase but also for suppression of C-NHEJ during M phase to prevent genome instability in human cells.

## Materials and Methods

### Plasmids

The plasmid containing the human XRCC4 gene was constructed as described [29]. The siRNA-resistant XRCC4 and XRCC4-AP (containing S326A substitution) constructs were generated by the introduction of three silent mutations in the XRCC4 siRNA–targeting region. The XRCC4-AP and the silent mutations were introduced by the *Dpn*I method [59]. To construct N-terminal FLAG-tagged XRCC4 and -XRCC4-AP, the 3×FLAG coding sequence was inserted at the 5′ end of the XRCC4 gene and then cloned into the *Eco*RV and *Bam*HI sites of pIRESpuro3 (Clontech). The resulting plasmids were named pMT285 and pMT400, respectively.

### Cell Lines

HeLaS3 cells were provided by the Riken Bio-Resource Center through the National Bio-Resource Project of the Ministry of Education, Culture, Sports, Science and Technology (MEXT) of Japan. GFP-histone H2B HeLa cells [60] and HeLaS3 cell lines were cultured in standard Minimum Essential Medium (MEM, 11095, Gibco), and HCT116 cells were cultured in McCoy's 5A Medium (16600, Gibco), both supplemented with 10% fetal bovine serum (JRH Biosciences). Stable cell lines expressing either FLAG-XRCC4 or FLAG-XRCC4-AP were selected and cultured in medium supplemented with 0.25 µg/ml puromycin (Wako). The siRNA-resistant FLAG-XRCC4- and FLAG-XRCC4-AP-expressing stable cell lines were established via transfection of HeLaS3 cells with pMT285 and pMT400, respectively. Therefore, the XRCC4 siRNA disrupts endogenous expression, but not exogenous expression, permitting phenotype analysis of the mutant protein. Transfection of plasmids was carried out using Lipofectamine 2000 (Invitrogen).

### Cell Cycle Synchronization

For double thymidine block, HeLaS3 cells were cultured in MEM containing 2.5 mM thymidine for 16 h, washed with phosphate-buffered saline (PBS), released in MEM without thymidine for 9 h, and then incubated in MEM containing 2.5 mM thymidine for 16 h. For thymidine nocodazole block, HeLaS3 cells were cultured in MEM containing 2.5 mM thymidine for 24 h, washed with PBS, released for 4 h, and then incubated in MEM containing 0.1 µg/ml nocodazole for 16 h.

### Preparation of Antibodies

The following antibodies were used for western blotting or immunoprecipitation: anti-XRCC4 (mouse, 611506, BD), anti-CtIP (sc5970, Santa Cruz Biotechnology), anti-XRCC3 (sc53471, Santa Cruz Biotechnology), anti-XLF (ab33499, Abcam), anti-tubulin (sc5286, Santa Cruz Biotechnology), anti-phospho-histone H3 (S10) (06-570, Millipore), anti-FLAG (1E6, Wako), anti-DNA ligase IV (this study) and anti-pS326 of XRCC4 (this study). Antibodies used for immunostaining were as follows: anti-53BP1 (NB100-304, Novus Biologicals), anti-Rad51 [61], anti-XRCC4 (this study), and anti-DNA ligase IV (this study). For preparation of antibodies against XRCC4 and DNA ligase IV, full-length human XRCC4 tagged with hexahistidine and a 367-residue peptide containing the C-terminus of human DNA ligase IV tagged with hexahistidine were affinity purified from *Escherichia coli* with a nickel/cobalt column and used for immunization of rat or guinea pig, respectively. Anti-pS326 was raised in rabbits against a synthesized phosphopeptide, TLRNSpSPEDLFC. Post-immune IgG was affinity purified with this phosphopeptide and also titrated using a non-phosphorylated peptide, TLRNSSPEDLFC (custom-made by MBL Co., Ltd.). Immunization and preparation of antisera were carried out by MBL Co., Ltd.

### Induction of M-phase DSBs and Detection of Anaphase Bridges

HeLaS3 cells grown on 13 mm–diameter round cover glasses (Matsunami) were arrested at M phase by treatment with 0.1 µg/ml nocodazole (Wako) for 3 h. Etoposide (Sigma-Aldrich) or neocarzinostatin (NCS, Sigma-Aldrich) or ICRF-159 (Sigma-Aldrich) was added to the culture medium to a final concentration of 10 µM, 1 ng/ml, 10 µM, respectively. Cells were incubated for 15 min in medium containing the drug and then washed twice with PBS. For detection of anaphase bridges, etoposide-treated cells were incubated in fresh culture medium for 1 h, fixed with 4% (w/v) paraformaldehyde (Sigma-Aldrich), and stained with 4′,6-Diamidino-2-Phenylindole (DAPI). The frequency of cells with at least one bridge was calculated by dividing the number of cells containing bridges by the number of total anaphase cells.

### Preparation of Metaphase Spreads for Assessment of Chromosome Aberrations

Cells were arrested in M phase by incubating with 0.1 µg/ml nocodazole for 45 min. Cells were trypsinized, washed with PBS, and then incubated in a hypotonic solution (0.05 M KCl) for 20 min at 37°C. Fixative (methanol/acetic acid glacial 3∶1, v/v) was added to a final concentration of 40%. The cells were washed twice with the fixative, then incubated for 30 min in the fixative, washed again with the fixative, and then surface-spread on a glass slide. Chromosomes were stained with 4% Giemsa (Merck) for 30 min. Spreads were observed under light a microscope with 63× objective (Zeiss).

### Comet Assay

HeLaS3 cells were arrested by the double thymidine block method at early S phase, washed twice, and then released for resumption of growth. After 6 h, nocodazole was added to a final concentration of 0.1 µg/ml and cells were incubated for 3 h to arrest during M phase. Mitotic cells were collected by mechanical shake-off. After treating the mitotic cells with a final concentration of 10 µM of etoposide for 15 min, the cells were washed twice with PBS, and further incubated in culture medium without the drug for various times. The neutral comet assay was performed using a Comet Assay kit (4250-050-K, Trevigen). HeLaS3 cells were subjected to comet analysis. Cells were embedded in low melting point agarose on a glass slide, lysed and subjected to electrophoresis at 1 V/cm for 20 min. After staining the cells with SYBR green, comet images were captured by fluorescence microscopy (AxioPlan; Zeiss). An average Comet-tail moment ([percentage of DNA content in tail]×[tail length]) was scored for more than 70 nuclei at each time point using CometScore software (TriTek).

### FACS

For analysis of cell-cycle progression, cells were permeabilized with 0.2% (v/v) Triton X100 in PBS, and then treated with 500 µg/ml RNase A (Nacalai Tesque) and 25 µg/ml propidium iodide. The cells were analyzed with a FACSCalibur flow cytometer with BD CellQuest Pro software (BD Bioscience). The cell-cycle phases were identified on the basis of their DNA content by propidium iodide staining.

### Immunoprecipitation and Western Blotting

HeLaS3 cells were washed in wash buffer (50 mM HEPES pH 7.5, 1 mM EDTA, 150 mM NaCl, 1 mM DTT), pelleted, resuspended in 1 ml of lysis buffer (50 mM HEPES pH 7.5, 1 mM EDTA, 150 mM NaCl, 1 mM DTT, 1% (v/v) NP40 and 0.5% (w/v) sodium deoxycholate, 1 mM sodium orthovanadate, 60 mM β-glycerophosphate) containing a protease inhibitor cocktail (25955-11, Nacalai Tesque) and 1 mM PMSF and lysed for 30 min on ice. After centrifugation at 20,000× *g* for 10 min, the supernatant was collected and incubated for 3 h at 4°C with 50 µl anti-mouse IgG–conjugated Dynabeads (Veritas) that had been pre-incubated with 2 µg mouse anti-FLAG for 3 h at 4°C. Immunoprecipitates containing FLAG-tagged XRCC4 were collected by magnetic capture and washed three times with 1 ml lysis buffer. Immunoprecipitates were treated with 10 U of calf intestinal phosphatase (New England BioLabs). Proteins in the immunoprecipitates were separated by SDS-PAGE (8% polyacrylamide gels) and transferred to a polyvinylidene fluoride membrane (Immobilon-P, Millipore). Western blotting was performed as described [30].

### Cytological Analysis by Indirect Immunostaining and Live-Cell Imaging

HeLaS3 cells grown on 13-mm-diameter round cover glasses (Matsunami) were arrested by nocodazole for 3 h, treated with or without etoposide for 15 min, and incubated for 1 h in culture medium without the drug. The cells were fixed with 4% paraformaldehyde for 20 min and then permeabilized with 0.2% (v/v) Triton X-100 for 10 min. Indirect immunostaining was performed as described [62] except that 5% bovine serum albumin in PBS used as the blocking reagent. Stained samples were observed using an epifluorescence microscope (AxioPlan with a 63× objective, NA1.4; Zeiss) equipped with a CCD camera (Retiga, QImaging). Images were processed using IP Lab (Scanalytics Inc.) and Photoshop (Adobe) software. Live-cell imaging was performed using GFP-histone H2B–expressing HeLa cells. For live-cell imaging analysis, cells were arrested at early S phase via double thymidine block, washed twice with fresh culture medium, and then released for resumption of growth. After 7 h, nocodazole was added to a final concentration of 0.1 µg/ml, and cells were incubated for 3 h to arrest during M phase. Mitotic cells were collected by mechanical shake-off. After treatment of mitotic cells with etoposide or DMSO (control) for 15 min, the cells were washed twice with PBS. Prometaphase cells were then chosen and imaged automatically using a time-lapse live-cell imaging system (Delta-vision; GE Healthcare) with a 60× objective (Olympus, PlanApo NA1.42) equipped with a CCD camera (Cool Snap, Roper). GFP-labeled chromatin was observed every 2 min for 3 h. A set of images from eight focal planes with 2.5-µm intervals was taken at each time point.

### siRNA Knockdown

HeLaS3 cells were transfected with siRNA using Lipofectamine RNAiMax (Invitrogen) for 72 h. The siRNAs used for depletion of XRCC4, CtIP, and XRCC3 have been reported previously, as follows; XRCC4 siRNA [47], AUAUGUUGGUGAACUGAGAdTdT; CtIP siRNA [63], GCUAAAACAGGAACGAATCdTdT; siXRCC3#1 [64], CAGAAUUAUUGCUGCAAUUAAdTdT; siXRCC3#2 [64], CAGCCAGAUCUUCAUCGAGCAdTdT;. HeLaS3 cells were transfected with the MISSION siRNA Universal Negative Control (Sigma-Aldrich) as a transfection control. All siRNAs were synthesized by Sigma Genosys.

## Supporting Information

Figure S1 Anaphase bridge formation in NCS- or ICRF-159-treated cells. (A) Anaphase bridge formation in non-etoposide-treated cells without nocodazole arrest (Noc−), non-etoposide-treated cells after nocodazole arrest (Noc+, Etp−), and etoposide-treated cells after nocodazole arrest (Noc+, Etp+). Error bars show the standard deviation from at least three independent experiments. (B) Anaphase-bridge formation in non-etoposide-treated (Noc+, Etp−) and etoposide-treated (Noc+, Etp+) HCT116 cells. Error bars show the standard deviation from at least three independent experiments. (C) Anaphase-bridge formation in non-treated (Noc+, NT), etoposide-treated (Noc+, Etp), ICRF-159-treated (10 µM; Noc+, ICRF-159), and NCS-treated (1 ng/ml; Noc+, NCS) cells. Anaphase bridge formation frequency was calculated in GFP-histone H2B–expressing cells by time-lapse imaging after 15 min incubation with the indicated drugs. For continuous ICRF-159 treatment, M-phase cells were incubated with ICRF-159 (10 µM) and observed for 6 h (continuous-ICRF-159), and anaphase bridge formation frequency was determined in each case. (D) Anaphase-bridge formation in HeLaS3 cells transfected with an siRNA targeting XRCC4, CtIP, or XRCC3 (or control siRNA) with (+NCS) or without (−NCS) induction of mitotic DSBs by NCS. Anaphase cells (≥50) were scored for each experiment.
(EPS)

Figure S2 53BP1 and Rad51 are excluded from DSB sites on mitotic chromosomes. (A) Localization of Rad51 (green; anti-Rad51) and γH2AX (red; anti-γH2AX) was analyzed in non-treated (NT) or mitotic DSBs–induced cells (Etp) at 1 h after release from etoposide treatment. Scale bar, 10 µm. (B) Frequencies of Rad51-positive (black bars) and γH2AX-positive (gray bars) chromosomes/nuclei in non-treated (NT) and etoposide-treated (Etp) cells at 1 h after release from etoposide treatment. (C) Localization of 53BP1 (green; anti-53BP1) and γH2AX (red; anti-γH2AX) was analyzed in non-treated (NT) or mitotic DSBs-induced cells (Etp) at 0, 1 and 2 h after release from nocodazole arrest. Scale bar, 10 µm. (D) Frequencies of 53BP1-positive (black bars) and γH2AX-positive (gray bars) chromosomes/nuclei in non-treated (NT) and etoposide-treated (Etp) cells at the indicated times after release from nocodazole arrest.
(EPS)

Figure S3 Anaphase bridge formation values in etoposide-treated cells compared after subtracting the value for the non-treated cells. (A) The frequency of anaphase bridge formation observed in etoposide-treated cells was corrected by subtracting the frequency measured for the non-treated cells shown in Figure 3B. Error bars show the standard deviation from three independent experiments. (B) The frequency of anaphase bridge formation observed in etoposide-treated cells was corrected by subtracting the frequency measured for the non-treated cells shown in Figure 5E. Error bars show the standard deviation from seven independent experiments. (C) Anaphase bridge formation observed by time-lapse imaging without etoposide treatment in CtIP knockdown cells. Data shown are for control siRNA (siControl) cells and CtIP knockdown (siCtIP) cells with or without etoposide treatment as shown in Figure 3D. Prometaphase cells (≥25) were chosen and analyzed for anaphase bridge formation for each experiment. Error bars show the standard deviation from four independent experiments. Statistical significance was analyzed with the Student's *t*-test. ** *P*-value<0.01, * *P*-value<0.05.
(EPS)

Figure S4 Expression of XRCC4 proteins in FLAG-XRCC4 and FLAG-XRCC4-AP cell lines and localization of endogenous XRCC4 and DNA ligase IV. (A) FLAG-XRCC4 or FLAG-XRCC4-AP, and endogenous XRCC4 proteins were detected by western blotting with anti-XRCC4 in HeLaS3 cells, and in the siRNA-resistant FLAG-XRCC4- and FLAG-XRCC4-AP-expressing stable cell lines in the presence of control siRNA or XRCC4 siRNA. Molecular weight markers are shown on right. (B) Indirect immunostaining of DNA ligase IV (green; anti-DNA ligase IV) and XRCC4 (red; anti-XRCC4) in non-treated (NT) and etoposide-treated (Etp) cells at 1 h after release from nocodazole arrest.
(EPS)

Figure S5 Efficiency of DSB repair in FLAG-XRCC4 and FLAG-XRCC4-AP cell lines. (A) An independent experiment of DSB repair kinetics in the XRCC4 and XRCC4-AP cells shown in Figure 5D. (B) Kinetics of average relative tail moment values in the etoposide-treated cells of each cell line shown in panel A.
(EPS)

Table S1 Chromosome aberration analysis in HCT116 cells.
(DOCX)
