# Supplementary material for: Canonical Non-Homologous End Joining in Mitosis Induces Genome Instability and Is Suppressed by M-phase-Specific Phosphorylation of XRCC4
Source: PLoS Genet. 2014 Aug 28;10(8):e1004563. doi: 10.1371/journal.pgen.1004563 (PMC4148217; doi:10.1371/journal.pgen.1004563)
Supplement: Table S1 — Chromosome aberration analysis in HCT116 cells. (DOCX) [file pgen.1004563.s006.docx]

**Table S1. Chromosome aberration analysis in HCT116 cells**

|  | –Etp | +Etp | *P*-value ^a^ |
| --- | --- | --- | --- |
| Fragmented chromosomes | 1 | 22 | <0.0001 |
| Ring chromosomes | 0 | 3 | 0.12 |
| Dicentric chromosomes | 0 | 2 | 0.25 |
| Metaphase cells analyzed (N) | 29 | 30 |  |

^a^ The significance of each difference between the non-treated (–Etp) and etoposide-treated (+Etp) cells was determined using Fisher’s exact test.
